# Comparison of the temporal and technical-tactical characteristics in badminton men’s singles under different competition formats

**DOI:** 10.3389/fpsyg.2025.1634776

**Published:** 2025-09-12

**Authors:** Yichang Zhao, Aiqing Zhu, Siyue Zhang, Yibo Zhang

**Affiliations:** School of Physical Education and Health Engineering, Taiyuan University of Technology, Taiyuan, China

**Keywords:** badminton, men’s singles, different competition formats, time structure, technical and tactical analysis

## Abstract

**Introduction:**

This study compared temporal structures, technical–tactical applications and scoring patterns between men’s singles matches played under two formats at the 2024 BWF World Youth Championships: an individual event (21-point system) and a team event (11-point relay system).

**Methods:**

A total of 40 matches were analyzed, with 20 matches for each competition format. Each match consisted of two sets, resulting in a total of 80 sets containing 22,283 batting actions, and each batting action was treated as an independent analytical unit.

**Results:**

Team matches were substantially shorter in overall duration, with large reductions in total playing time, rest intervals and whole-game length. In contrast, no significant differences emerged for rally-level indicators—shots per rally, time per stroke or rest between rallies—indicating that competitive intensity within rallies remained comparable. Technically, individual matches featured more net-based strokes such as spinning net shots and lifts to create attacking chances, whereas team matches favored safer choices like drop shots and interceptions. Scoring patterns diverged accordingly: unforced errors dominated in individual matches, whereas forced errors were more prevalent in the team event.

**Discussion:**

The 11-point relay system therefore maintains rally intensity while shortening total match time, lessening physical load and heightening psychological demands. The findings offer empirical guidance for youth competition reform and coach education.

## 1 Introduction

The systematic establishment of modern badminton rules can be traced back to 1870, when the Duke of Beaufort in the UK formulated the basic framework of badminton game rules. In 1893, after the establishment of the Badminton Association of England, the first complete set of competitive rules was officially promulgated in document form. Since then, the badminton sport has undergone multiple institutional and rule reforms. In 1992, badminton became an official Olympic event, adopting a three-game-two-win system with a 15-point scoring rule and a service-right-based scoring mechanism, where points could only be accumulated after obtaining the service right and continuously winning rallies. However, this rule was controversial due to excessively long match durations. In 2006, the International Badminton Federation introduced the current 21-point three-game-two-win system, abolishing the service-right-based scoring rule, which has been used ever since.

Since the implementation of the new rules, research on the competitive aspects of badminton has become more systematic. Many studies have analyzed badminton through its time structure, as different time structures influence athletes’ energy supply (Wang and Guo, 2023). Among these studies, the most extensive research focuses on Olympic competitions. Some researchers compared six Olympic tournaments, analyzing rally duration, rest intervals, the number of strokes per rally, and stroke frequency, revealing significant fluctuations in time structure. The shot frequency demonstrates a 34.0% increase (*p* < 0.000001; Ƞ^2^ = 0.17), while work density exhibits a 58.2% decrease (from 78 to 30.8%). Additionally, the effective playing time shows a reduction of 34.5% (from 34.7 ± 1.4% to 22.7 ± 1.4%) (Laffaye et al., 2015). Time structure can effectively reflect changes in badminton competition systems and rules. Within the same Olympic tournament, all time-related indicators in men’s singles and women’s singles knockout stages are higher than those in group stages (Torres-Luque et al., 2019), indicating that knockout matches are more intense under the same competition conditions (Chiminazzo et al., 2018). Through time feature analysis, it is evident that athletes exhibit stronger aggressiveness under the new scoring system (Chen and Chen, 2008).

In addition to time structure, scoring structure is another key focus of many studies. During each stage of a match, winners consistently maintain a larger point differential than losers. The winners maintained a minimum five-point advantage over their opponents from the midpoint to the end of the game (Barreira et al., 2017). Therefore, some researchers have developed models to evaluate the impact of point differentials established by athletes at different stages on match outcomes. Provide easily observable and quantifiable scientific data, and confirm that the outcome of a match is largely determined once the leading score exceeds five points (Wang, 2022).

Tactical analysis is a major issue in badminton, and the ability to better utilize tactics is crucial for achieving victory (Chu et al., 2022). Some studies have investigated differences in matches between athletes with different handedness, finding that variations in technical-tactical application are the primary distinctions between such athletes. The primary effect of the left-handed player on the right-handed opponent’s performance was a reduction in overhead strokes, an increase in drive shots, a predominance of small slashes, and a decrease in large slashes (Gomez et al., 2019; Zhang and Leng, 2024). In terms of technical-tactical application, there are numerous studies on the first and last strokes, reflecting an athlete’s strategic choices and primary scoring methods. The gold medalist primarily used backhand and forehand flick serves, especially backhand short serves in sets 1 and 2 across all tournament stages. The bronze medalist preferred forehand long serves throughout the match, while the silver medalist employed a mixed serving strategy combining forehand short, backhand short, and forehand long serves. Scoring and losing points were most common in rallies lasting 1–8 shots (20.76 and 32.99%, respectively), followed by 9–16 shots, with the lowest frequency in rallies exceeding 16 shots, indicating the early phase is critical in match outcomes (Gomez-Ruano et al., 2020; Sheng et al., 2021). The usage rates of various techniques and research on the techniques themselves have also become important indicators for tactical analysis in badminton (Chen et al., 2019; Abedanzadeh et al., 2025).

Following the reform to the 21-point system, some researchers discovered trends toward longer rest intervals in contemporary matches, reducing the expected effects of the rule change (Hoffmann and Vogt, 2023). Others analyzed technical-tactical characteristics under different scoring systems, finding significant differences in strategy selection and technical usage post-reform. After the competition format changed, athletes increased offensive efforts, resulting in a 6% decrease in push shot usage. Although block shot usage rose by 3%, the scoring rate dropped by 22%. During net defense, successful players increased blocking to create attacking opportunities. Mid-court spike shots remained the most frequently used technique, with an average usage rate of 58%, and their usage pattern was unaffected by the format change. These findings highlight the importance of analyzing the impact of different scoring systems on playing strategies (Zhong, 2010; Zhou and Dai, 2010).

This study built upon existing literature by adopting the three-phase temporal structure proposed by Fu and Cheng (2020) which categorizes rally durations into three intervals: 0–10 s, greater than 10 s, and greater than 20 s of rest between rallies. The “round rhythm coefficient” was employed as a key metric to assess the stability of match intensity. With regard to technical-tactical variables, this research integrated the coding frameworks developed by Chu et al. (2022) and Sheng et al. (2021), classifying stroke techniques into ten subcategories across three court zones—net, midcourt, and backcourt. Additionally, stroke direction and landing zone distributions were incorporated to better characterize athletes’ spatial control strategies. The men’s singles event at the 2024 World Youth Championships was selected as the research context due to its unique implementation of both the 21-point individual scoring system and the newly introduced 11-point team relay format within the same cohort of athletes. This novel 11-point relay system, implemented for the first time in the 2024 World Junior Championships, aims to reduce match duration and enhance spectator engagement. However, no empirical analyses have yet been conducted on the technical-tactical characteristics of this format. The concurrent application of both scoring systems within the same competition setting effectively controls for athlete skill variability and provides a rare quasi-experimental scenario to investigate the causal relationship between competition format and athletic performance.

This study is designed to conduct a rigorous examination of the complex interplay between the evolving regulatory environment and the progressive advancement of technical-tactical innovations. Through the systematic integration of temporal dynamics with the multidimensional aspects of technical and tactical performance, the research aims to produce robust empirical results that can serve as a foundation for the systematic evaluation of newly implemented regulations. Furthermore, the study intends to develop a comprehensive theoretical framework intended to enhance the accuracy and efficacy of athletes’ tactical planning and strategic decision-making processes within competitive contexts.

## 2 Methods

### 2.1 Participants and general procedure

This study included 20 matches from the knockout stage of the men’s singles event in the 2024 World Youth Championships and another 20 matches from the knockout and placement stages of the men’s singles in the team event of the same championship. A total of 40 matches were analyzed, with 20 matches for each type of competition format. Each match consisted of two sets, resulting in a total of 80 sets containing 22,283 batting actions. Each batting action was treated as an independent analytical unit. Each stroke performed by an athlete was considered an observation unit. Video analysis software was utilized to capture frame pauses and record rally durations, rest intervals, and total match duration.

This study focuses on the men’s singles event of the 2024 World Youth Championships. The research sample comprises 20 matches selected from the knockout stage of the individual men’s singles competition, as well as an additional 20 matches from both the knockout and qualifying stages of the men’s singles team event. The inclusion of matches from these stages was intended to ensure a high standard of competition and data reliability.

All participating athletes were male junior badminton players aged between 17 and 18 years. As all matches were drawn from the same championship, it was ensured that each athlete participated in both the individual and team formats of the event.

In this study, each stroke executed by an athlete was treated as an independent observational unit. Relevant data were collected using video analysis software, through which frame-by-frame playback was utilized to record key temporal metrics, including rally duration, rest intervals, and total match duration.

### 2.2 Data collection

The independent variable in this study was the format of the competition (individual versus team events). The dependent variables included match scores and stroke outcomes, specifically the total points and number of strokes per match. Additionally, the study examined the percentages of rally outcomes, positions, techniques, trajectories, and landing points (with detailed definitions provided in Tables 1–9). Furthermore, all standardized technical terminology utilized in this study was sourced from the author’s prior research (Zhang and Leng, 2024). All data in this study were coded by a chief analyst with over 10 years of experience in badminton technique and tactical analysis. To ensure coding reliability, a reliability test was conducted prior to the formal experiment. Three matches (a total of seven sets) were randomly selected for double verification: the chief analyst independently re-coded the same matches after a 72-h interval, and a second senior analyst independently performed parallel coding. Both infra-observer and inter-observer re-liabilities were assessed using Cohen’s *κ* coefficient. The results showed κ values exceeding 0.85 for all variables, indicating strong agreement and meeting the acceptable reliability standard. In cases where κ was below 0.85 or coding discrepancies occurred, the two analysts jointly reviewed the match videos and discussed until a consensus was reached. If necessary, the project leader provided final arbitration and updated the final coding accordingly.

**Table 1:** All variables and their meanings.

Dimension	Variable	Definition and formula	Unit/note
Time	Average shot per rally	Total number of strokes ÷ Total number of rallies (Abian-Vicen et al., 2013)	Strokes·rally^−1^
Time	Average time per shot	Total stroke duration ÷ Total number of shots (Abian-Vicen et al., 2013)	s·shot^−1^
Time	Average time of intervals	Total time for intervals / Total number of intervals (Abian-Vicen et al., 2013)	s·interval^−1^
Time	time structure	Rallies in a given phase ÷ Total rallies ×100 %Phases: 0–10 s rallies, >10 s rallies, ≥20 s rest (Sheng and Dai, 2015)	%
Time	Rally rhythm coefficient	Strokes per rally ÷ Rally duration ×100% (Fu and Cheng, 2020)	Strokes·s^−1^
Tactics and techniques	Technical usage rate	Number of technical executions ÷ Total number of strokes × 100% (Zhang and Leng, 2024)	%
Tactics and techniques	Technical scoring/losing rate	Number of scoring/losing events using a specific technique ÷ Total number of technical executions × 100% (Zhang and Leng, 2024)	%
Space	Hitting route usage rate	Number of times a specific line is used ÷ Total number of line usages × 100% (Zhang and Leng, 2024)	%
Space	Landing point usage rate	Number of times a specific landing point is used / Total number of landing point usages × 100% (Zhang and Leng, 2024)	%
Scoring	The nature of points gained and lost	Proportion of scoring/losing events attributed to a specific factor Total number of scoring/losing events × 100% (Zhang and Leng, 2024)	%

**Table 2:** Standardized nomenclature for tactical elements.

Technical and tactical name
Spinning shuttle and net drop
Lift
Flick
Cross-court net
Kill and brush
Block
Drive
Intercept
Clear
Drop
Smash

**Table 3:** Comparison of temporal characteristics: individual matches exhibit longer total time, shorter rally durations, and extended intervals compared to team matches.

Time characteristic indicators	Individual	Team	*z*	*p*	*d*
Average time per shot	8.80 ± 1.73	8.56 ± 1.62	−0.592	0.554	0.133
Total time for shots	320.67 ± 79.50	170.52 ± 50.30	−6.88	<0.001	2.408
Average time of intervals	21.41 ± 4.48	20.42 ± 4.59	−0.938	0.348	0.211
Total time for intervals	789.31 ± 316.32	401.77 ± 195.13	−6.418	<0.001	2.06
Average shot per rally	10.05 ± 2.21	9.57 ± 1.61	−0.818	0.413	0.184
Total time for the game	1123.37 ± 428.98	590.31 ± 214.23	−6.437	<0.001	2.073

**Table 4:** Temporal structure comparison: individual matches show higher proportion of >10-s rallies and >20-s rests than team matches.

Time structure indicators	Individual	Team
Rally duration 0–10 s	69.09	70.92
Rally duration >10 s	30.91	29.08
Rest duration >20 s	0.42	0.35

**Table 5:** Comparison of rhythm coefficients: individual matches display greater rally rhythm coefficient than team matches.

Rhythm coefficient indicator	Individual	Team	*z*	*p*	*d*
Rally rhythm coefficient	1.161	1.122	4.426	<0.01	0.18

**Table 6:** Comparison of particular techniques: individual matches favor spinning net shots and lifts, while team matches emphasize drops and intercepts.

Techniques	Individual	Team	*z*	*p*	*d*
Spinning shuttle and net drop	0.08 ± 0.04	0.06 ± 0.04	−3.049	0.002	0.497
Lift	0.20 ± 0.05	0. 18 ± 0.06	−2.530	0.011	0.408
Flick	0.20 ± 0.06	0.21 ± 0.06	−0.978	0.328	0.155
Cross-court net	0.06 ± 0.03	0.06 ± 0.04	−0.445	0.656	0.070
Kill and brush	0.03 ± 0.02	0.03 ± 0.02	−2.413	0.016	0.389
Block	0.02 ± 0.01	0.02 ± 0.02	−0.640	0.522	0.101
Drive	0. 11 ± 0.03	0. 12 ± 0.04	−1.560	0.119	0.249
Intercept	0.05 ± 0.03	0.04 ± 0.03	−2.041	0.041	0.327
Clear	0.02 ± 0.01	0.01 ± 0.01	−2.844	0.004	0.461
Drop	0.03 ± 0.02	0.05 ± 0.04	−3.451	0.001	0.567
Smash	0.08 ± 0.04	0.09 ± 0.05	−0.433	0.665	0.069

**Table 7:** Comparison of the nature of points gained and lost: individual matches yield more forced errors, team matches record higher unforced errors.

Rally outcome	Individual	Team	*z*	*p*	*d*
Forced error	0.198 ± 0.093	0.270 ± 0.161	−2.883	0.004	0.468
Unforced error	0.300 ± 0.133	0.256 ± 0.140	−2.173	0.03	0.349
Active error	0.399 ± 0.152	0.353 ± 0.195	−1.743	0.081	0.278
Direct point	0.098 ± 0.070	0. 120 ± 0.110	−0.977	0.328	0.155

**Table 8:** Comparison of scoring rates of specific techniques: kill-and-brush shots achieve higher success in individual matches than in team matches.

Techniques	Individual	Team	*z*	*p*	*d*
Spinning shuttle and net drop	0.01 ± 0.02	0.01 ± 0.03	−1.159	0.247	0.184
Lift	0.01 ± 0.02	0.01 ± 0.03	−0.336	0.737	0.053
Flick	0.04 ± 0.07	0.07 ± 0.16	−0.085	0.933	0.013
Cross-court net	0.05 ± 0.15	0.05 ± 0.16	−0.007	0.995	0.001
Kill and brush	0.50 ± 0.32	0.26 ± 0.35	−4.232	<0.001	0.789
Block	0.01 ± 0.03	0.01 ± 0.03	−1.986	0.047	0.318
Drive	0.04 ± 0.08	0.06 ± 0.16	−0.279	0.780	0.045
Intercept	0.01 ± 0.04	0.00 ± 0.00	−0.820	0.412	0.155
Clear	0.03 ± 0.13	0.02 ± 0.07	−0.141	0.888	0.023
Drop	0.03 ± 0.16	0.03 ± 0.12	−2.008	0.045	0.323
Smash	0.18 ± 0.10	0.17 ± 0.14	−1.105	0.269	0.175

**Table 9:** Comparison of error rates of specific techniques: intercept errors are more frequent in individual matches than in team matches.

Techniques	Individual	Team	*z*	*p*	*d*
Spinning shuttle and net drop	0.05 ± 0.33	0.06 ± 0.06	−0.730	0.466	0.116
Lift	0.06 ± 0.05	0.06 ± 0.06	−0.061	0.952	0.010
Flick	0.09 ± 0.12	0.06 ± 0.11	−1.940	0.052	0.310
Cross-court net	0. 11 ± 0.18	0. 10 ± 0.21	−1.286	0.199	0.218
Kill and brush	0. 12 ± 0.19	0. 12 ± 0.30	−1.558	0.119	0.273
Block	0. 13 ± 0.09	0. 14 ± 0.13	−0.373	0.709	0.059
Drive	0.07 ± 0.12	0.08 ± 0.19	−1.213	0.225	0.197
Intercept	0.07 ± 0.17	0.02 ± 0.15	−2.757	0.006	0.54
Clear	0.08 ± 0.20	0.04 ± 0.14	−1.377	0.169	0.227
Drop	0.06 ± 0.10	0.07 ± 0.14	−0.760	0.447	0.121
Smash	0.09 ± 0.08	0.08 ± 0.09	−0.144	0.886	0.023

### 2.3 Materials

**Figure 1:**
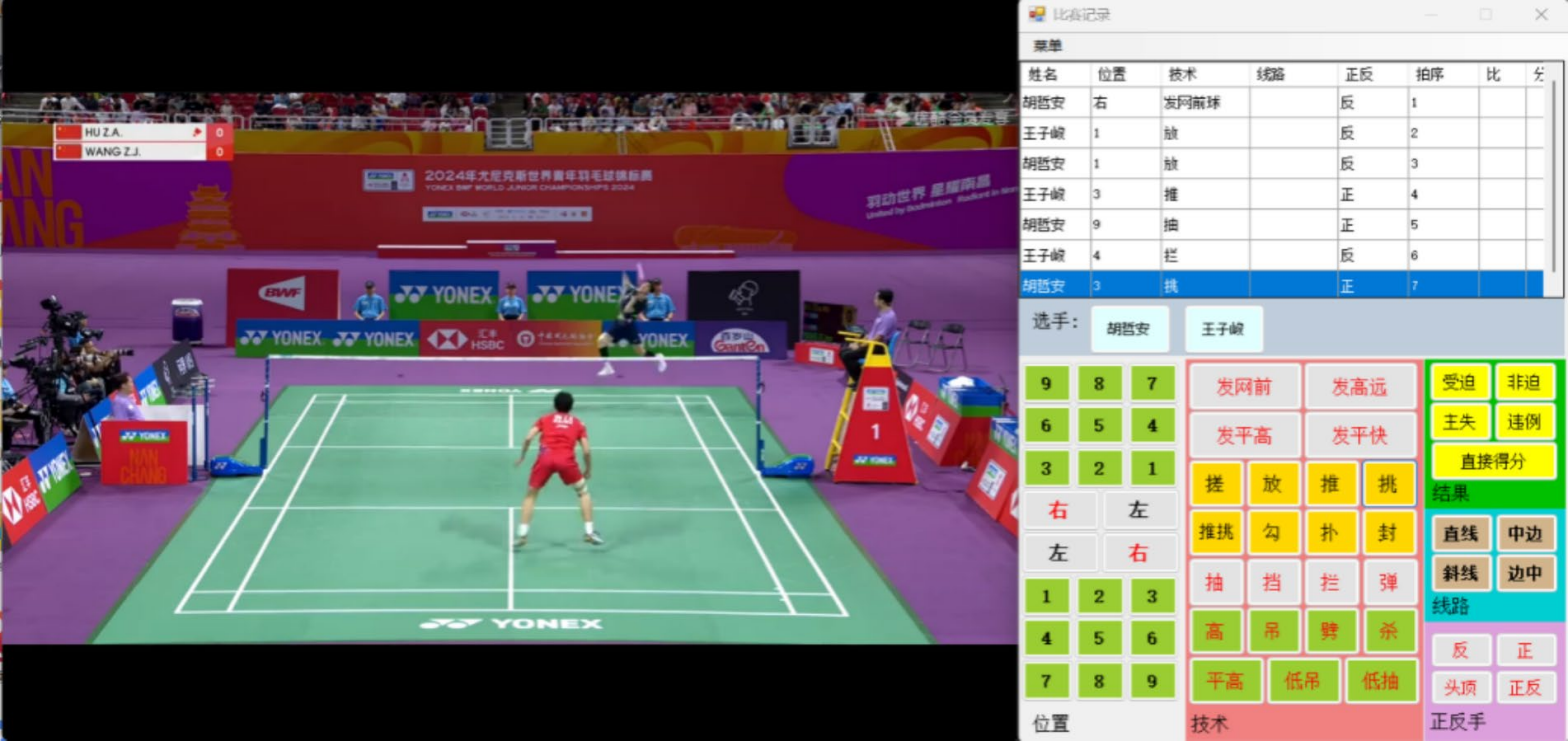
Badminton competition data collection system interface.

The official videos from the 2024 World Youth Badminton Championships, recorded by the Badminton World Federation (BWF), were utilized for match analysis in this study. It should be noted that all measurements and observations were conducted by a single investigator who had received extensive training in the study’s methodology and procedures. This individual was responsible for analyzing match videos and directly recording observations using a specialized badminton match information recording system. A dedicated badminton statistics program was employed to document technical characteristics (Zhang and Tang, 2023). To enhance data collection efficiency and accuracy, this study developed a badminton competition information collection system using computer programming technology. The system was implemented as a WinForm application based on the. NET framework with C#, incorporating visualization and event-driven programming techniques. It enables users to input data—such as hitting point, technique, and position—by clicking corresponding buttons, with all information automatically recorded in Excel tables. The system features an intuitive and user-friendly interface, integrating functions such as data entry, display, file management, and export. Once player names are specified in the menu bar, the program automatically alternates their names in the table according to the hitting sequence. Additionally, it calculates and records the hitting order in real time while capturing technical and positional details, scoring outcomes (win/loss), and rally length (number of strokes per rally). Each technical move was paused to allow video statisticians to confirm specific variables, ensuring accuracy and consistency in data collection. Therefore, when using this software, relevant variables can be adjusted according to the game situation. The study focused on comparing the performance of the same group of players in both individual and team events, assessing differences in their technical and tactical applications across the two formats. Figure 1 shows the interface of the badminton competition data collection system.

### 2.4 Statistical analysis

This study focused on the men’s singles badminton competition and used the systematic video observation method to collect data from 40 individual matches (21-point system) and 40 team matches (11-point system) in the 2024 World Youth Badminton Championships (total sample size *N* = 160, each match included two athletes), with each match serving as the analysis unit. Using Excel 2021, the frequency statistics of the number of racket strokes, technical categories, landing distribution, and line were conducted, and the percentage of each indicator’s technical performance was calculated. Given the presence of multiple variables and the failure of certain continuous indicators to meet the normality assumption as determined by the Shapiro–Wilk test, the non-parametric Mann–Whitney U test was consistently applied to assess all inter-group differences in this study. This approach was adopted to mitigate potential biases arising from violations of distributional assumptions and to ensure the robustness of statistical inference. The significance level was set at α = 0.05 (two-tailed), it should be emphasized that in this study, the term “significant” applies exclusively to individual variables, and no cross-variable or overarching generalizations will be drawn during the subsequent interpretation. Additionally, the Exact option in SPSS 25.0 was utilized to compute precise *p*-values, ensuring valid statistical inference even under conditions of limited sample size. To complement the statistical analysis with a measure of practical relevance, Cohen’s d effect size was calculated for all comparisons. Effect size thresholds were established according to conventional guidelines, with 0.20, 0.50, and 0.80 indicating small, medium, and large effects, respectively. This dual reporting approach facilitates the concurrent interpretation of both statistical and practical significance, thereby offering a scientifically grounded foundation for future training interventions and competition system optimizations. Consistent with previous studies in this domain, each variable associated with the two competition formats (individual event versus team event) was examined as a distinct research question. Accordingly, all metrics within the time, tactics and techniques, space, and scoring were analyzed separately, and inter-group differences were evaluated using individual Mann–Whitney U tests. No Holm–Bonferroni or other multiple-comparison corrections were applied.

The mean and standard deviation of different indicators in the (individual, team) competition systems were calculated. The mean represents the average level of a certain measurement indicator for different actions in the “individual” and “team” competition systems, while the standard deviation reflects the dispersion of the data around the mean.

The independent variable is the competition format (individual 21-point system vs. team 11-point relay system). The dependent variable is defined in four dimensions: time, tactics and techniques, space, and scoring. All the variables have been presented in Table 1, while the standardized terminology for tactics and techniques is provided in Table 2.

## 3 Results

### 3.1 Time characteristic results

Table 3 shows the temporal characteristics of the two competition formats. The total time exhibits significant differences. Specifically, the total ball-hitting time in team events is reduced by 46.8% compared to individual events (*p* < 0.001, *z* = −6.880, *d* = 2.408), the total interval time is shortened by 49. 1% (*p* < 0.001, z = −6.418, *d* = 2.060), and the overall duration decreases by 47.4% (*p* < 0.001, *z* = −6.437, *d* = 2.073). These effect sizes indicate large practical significance.

In terms of per-rally ball-hitting and interval times, all indicators show *p*-values greater than 0.05, indicating no statistically significant differences between individual and team events in terms of average number of strokes, average ball-hitting time, and average interval time. The effect size for average interval time approaches the threshold for a small effect (*d* = 0.211), while the effect sizes for differences in average rally count and ball-hitting time are minimal (*d* < 0.2).

### 3.2 Results of time structure and rhythm coefficient

Table 4 shows the proportion of rounds for the two competition formats under different time structures. After verification, it was found that the proportion of 0-10s rounds for both formats exceeded 69%. The proportion of 0–10 s rounds in the team event was slightly higher than that in the individual event by 1.83%. The duration of intervals longer than 20 s in the individual event was 7% higher than that in the team event.

Table 5 presents the rhythm coefficients of two different competition formats. The rhythm coefficient of the individual event is 1.161, while that of the team event is 1. 122 (*p* < 0.01, *t* = 4.426, *d* = 0. 18), showing a statistically significant difference.

### 3.3 Usage of tactics and techniques

Table 6 shows the usage of various techniques in two different competition formats. The following technical indicators exhibit statistically significant differences between the individual event and the team event. The frequency of Spinning shuttle and net drop is significantly higher in the individual event than in the team event by 2% (*p* = 0.002, *z* = −3.049, *d* = 0.497). The frequency of drop is significantly higher in the team event than in the individual event by 2% (*p* = 0.001, *z* = −3.451, *d* = 0.567). The frequency of clear is significantly higher in the individual event than in the team event by 1% (*p* = 0.004, *z* = −2.844, *d* = 0.461). Additionally, statistically significant differences are observed for lift (*p* = 0.011, *z* = −2.530, *d* = 0.408), block (*p* = 0.016, *z* = −2.530, *d* = 0.389), and set (*p* = 0.041, *z* = −2.041, *d* = 0.461). No statistically significant differences are found in the usage frequencies of flick, Cross-Court Ne, block, drive, and smash between the two formats (*p* > 0.05). Among these, the difference in smash is the smallest (*p* = 0.665, *t* = −0.433, *d* = 0.069).

### 3.4 Nature of gains and losses

Table 7 shows the nature of gains and losses under two different competition formats. Statistical analysis revealed that, compared between the two formats, the number of forced errors in the team event was significantly higher than in the individual event by 7.2% (*p* = 0.004, *z* = −2.883, *d* = 0.468). Conversely, the number of unforced errors in the individual event was significantly higher than in the team event by 4.4% (*p* = 0.03, *z* = −2.173, *d* = 0.349). No statistically significant differences were observed in other aspects of gains and losses.

### 3.5 Result of technical gain and loss rate calculation

Table 8 shows the technical scoring rates for two different competition formats. In terms of scoring rate indicators, significant differences are observed in the scoring rates of three techniques: kill and brush, block, and drop between individual and team events. Specifically, the kill and brush scoring rate in front-court techniques is 24% higher in individual events than in team events (*p* < 0.001, *z* = −4.232, *d* = 0.789). The mean scoring rate of block in midfield techniques is 0.01 ± 0.03 in both competition formats, yet the difference remains statistically significant (*p* = 0.047, *z* = −1.986, *d* = 0.318). Additionally, the scoring rate of drop in back-court techniques also exhibits a significant difference (*p* = 0.045, *z* = −2.008, *d* = 0. 121). No statistical differences are found in other indicators.

Table 9 shows the technical error rates for two different competition formats. Among the error rate indicators, intercept is the only technique with a statistically significant difference. The intercept error rate in the individual event is 5% higher than that in the team event (*p* = 0.006, *z* = −2.757, *d* = 0.54). Other indicators exhibit no statistically significant differences.

### 3.6 Hitting routes and landing points distribution

Table 10 shows the comparison of hitting routes under two different competition formats, revealing statistically significant differences in the following indicators: Route 3 exhibited a 1% increase in usage during individual events compared to team events (*p* = 0.025, *t* = −2.235, *d* = 0.359); Route 7 showed a 3% decrease in usage during individual events relative to team events (*p* = 0.008, *t* = −2.669, *d* = 0.432); and Route 8 demonstrated a 2% reduction in usage during individual events compared to team events (*p* = 0.026, *t* = −2.234, *d* = 0.359). No statistically significant differences were observed for the remaining routes.

**Table 10:** Comparison of hitting routes: individual matches prefer big-slash angles, team matches favor straight drives.

Routes		Individual	Team	*z*	*p*	*d*
Left court	Straight	0.19 ± 0.05	0.17 ± 0.07	−1.913	0.056	0.306
	Small slash	0.09 ± 0.04	0.10 ± 0.04	−0.428	0.668	0.068
	Big slash	0.09 ± 0.04	0.08 ± 0.03	−2.235	0.025	0.359
Right court	Straight	0.20 ± 0.05	0.18 ± 0.06	−1.623	0.105	0.259
	Small slash	0.06 ± 0.03	0.07 ± 0.03	−0.224	0.823	0.035
	Big slash	0.06 ± 0.03	0.06 ± 0.04	−0.898	0.369	0.142
Center court	Straight	0.14 ± 0.04	0.17 ± 0.07	−2.669	0.008	0.432
	Small slash	0.07 ± 0.03	0.09 ± 0.04	−2.234	0.026	0.359
	Big slash	0.09 ± 0.03	0.09 ± 0.05	−0.015	0.988	0.002

Table 11 shows the usage of landing points under two different competition formats, with significant differences primarily observed in the net area. Landing point 1 exhibited a 3% increase in usage during individual events compared to team events (*p* < 0.001, *t* = −4.430, *d* = 0.748), while landing point 3 demonstrated a 2% increase in individual events relative to team events (*p* = 0.016, *t* = −2.403, *d* = 0.387). Additionally, landing point 8 showed a significant difference, with a 1% decrease in usage during individual events compared to team events (*p* = 0.007, *t* = −2.699, *d* = 0.437). No statistically significant differences were observed for the remaining landing points.

**Table 11:** Comparison of landing points: individual matches target left front court more heavily, team matches distribute more centrally.

Landing points		Individual	Team	*z*	*p*	*d*
Force court	Left	0.19 ± 0.04	0.16 ± 0.05	−4.430	<0.001	0.748
	Center	0.17 ± 0.05	0.19 ± 0.07	−1.749	0.080	0.279
	Right	0.17 ± 0.05	0.15 ± 0.06	−2.403	0.016	0.387
Mid court	Left	0.07 ± 0.03	0.07 ± 0.03	−0.334	0.738	0.053
	Center	0.08 ± 0.03	0.08 ± 0.04	−1.496	0.135	0.238
	Right	0.07 ± 0.03	0.06 ± 0.03	−0.345	0.730	0.055
Back court	Left	0.11 ± 0.04	0.12 ± 0.05	−0.764	0.445	0.121
	Center	0.05 ± 0.03	0.06 ± 0.03	−2.699	0.007	0.437
	Right	0.09 ± 0.04	0.10 ± 0.05	−1.804	0.071	0.288

## 4 Discussion

### 4.1 Comparison of time characteristics under different competition formats

Research indicates that significant differences exist among the three key indicators: total time for shots, total time for interval, and total time for game. This finding confirms the substantial impact of the scoring system reform on the temporal structure of badminton matches (Cabello Manrique and González-Badillo, 2003). At a competitive level, badminton demands a high proportion of individual aerobic capacity, with athletes required to sustain this type of exercise for approximately 30 min (Liu et al., 2023). The reduction in total match duration decreases the time athletes must engage in high-intensity aerobic activity. Studies reveal that muscle fatigue increases the risk of injuries, such as ankle strains (Gómez et al., 2021). Under the 11-point team event system, the maximum physical load on athletes has diminished, potentially reducing the likelihood of sports-related injuries caused by excessive fatigue. However, the compressed match duration allows both players to respond to each stroke with relatively higher energy levels. Consequently, world-class male singles players who rely on physically demanding strategies, such as energy-consuming rallies, may find it challenging to leverage their strengths. Simultaneously, the heightened technical demands necessitate athletes to rapidly adapt and maximize their tactical performance within limited time frames. Previous research highlights that the initial 11 points represent the phase with the most significant score fluctuations (Nosaka and Chen, 2024), during which athletes familiarize themselves with opponents.

It has been proposed that success in badminton not only hinges on an athlete’s understanding of the game but also on their ability to make swift judgments at the outset (Barreira et al., 2017). The decreased total time for shots and total time for intervals impose greater psychological and adaptive challenges, particularly in the early stages where athletes have less time to assess opponents and make rapid decisions. In team events, athletes’ comprehensive skills become more critical compared to individual events. Some scholars suggest that the interval between matches is influenced by the game load; for instance, sports like football require longer recovery periods, whereas badminton typically allows only one day of rest (Nosaka and Chen, 2024). If the game load is excessively high while the interval remains unchanged, athletic performance may be compromised. The new competition format reduces the physical burden on athletes, ensuring that their performance is less affected by the fixed interval compared to individual events, thereby enhancing the entertainment value of matches.

The average number of rounds, the average time per shot, and the average time of intervals effectively characterize the temporal features of a match. Prior research has explored the differences in time systems between the 21-point and 15-point scoring systems (Ming et al., 2008), revealing that the overall average match duration between the two systems is not significantly different, and the active playing time remains unaffected by the variations in scoring systems. The findings of this study align with previous observations, indicating that changes in scoring systems exert minimal influence on athletes’ time-related characteristics within individual rallies. Additionally, some scholars have noted that a reduction in the average time per shot coupled with an increase in the average time of intervals can serve as an effective indicator of heightened match intensity (Gomez-Ruano et al., 2020). Based on these insights, it can be inferred that the intensity levels of the two scoring systems are comparable.

### 4.2 Comparison of time structure and rhythm coefficient

Through the analysis of the time structure, whether it is a individual match or a team match, short rallies occupy the majority, reflecting the trend of faster rhythm in the men’s singles project nowadays. However, the differences between the two events themselves are not significant. The number of rallies lasting more than 20 s in the team match has slightly increased, indicating that after the emergence of the multi-shot hold stage, team players will be more cautious and extend the overall game duration.

Studies have found that the current badminton time structure presents a new trend. The rhythm of hitting the ball has increased, and the duration of hitting the ball has also been prolonged (Laffaye et al., 2015). In terms of the rhythm of hitting the ball, some scholars have proposed that the shortening of the scoring system will accelerate the game rhythm and increase the intensity of the game. As mentioned above, the average number of hits, the average duration of hitting the ball, and the average duration of intervals are slightly lower in the team match than in the singles match, and there is no significant difference. In terms of rhythm, the singles match is faster than the team match. It is found that the change in the scoring system does not increase the rhythm of the game but reduces it. Different from the traditional 11-point five-set best-of-three system, applying the 11-point relay system to the team match increases the responsibility of players and the pressure on them. At the same time, because the scoring system and the space for scoring and chasing points are shortened from 21 points to 11 points, athletes are relatively conservative in their playing style and do not rush to improve speed. Players will slow down the game while maintaining stability and reducing errors. The choice of tactics has shifted from “efficiency priority” to “stability priority.”

### 4.3 The difference in the application of techniques and tactics

In singles badminton matches, players typically prepare for the decisive shot only after employing a variety of hitting techniques and positioning strategies (Tong and Hong, 2000). Some scholars have suggested that the techniques in other court positions primarily serve to facilitate the final smash. The author posits that the most effective technique for creating opportunities for smashes or downward pressure is net play. In individual events, net-based techniques such as spinning shuttle and net drop are utilized more frequently, with the aim of gaining net control and forcing opponents to lift the shuttlecock, thereby creating offensive opportunities. Individual events exhibit a stronger proactive approach in generating offensive chances compared to team events, which prioritize stability and reduce the use of intricate net-based techniques to minimize risks. Because using control net-prevention techniques such as spinning shuttle and net drop would lead to more mistakes, and also because the quality of the ball thrown would not be sufficient, it would result in the opponent directly using kill and brush and other techniques to score. As for the attacking ball technique, which is an active starting ball technique, it is used more in individual competitions. This shows that in team competitions, the tactical options lie more in controlling the game to avoid starting balls as much as possible while maintaining stability.

When holding an advantage in the backcourt, team events tend to use fewer high clears and more drop shots. Drop shots are less prone to errors compared to smashes and also serve a strategic purpose of maneuvering the opponent. This highlights the enhanced strategic nature of team events and the greater emphasis on maneuvering opponents. In passive situations, team events rely more on Interception techniques rather than lifting high clears to invite attacks from opponents. Interception, similar to drop shots, helps maintain a downward pressure state. In contrast, individual events feature a broader range of techniques, including frequent use of net-side flicks and backcourt clears, resulting in larger playing spaces and increased movement ranges for athletes.

### 4.4 The nature of scoring and losing points, and the scoring/losing rates of specific techniques

After research, it has been found that in both singles and team matches, the scoring method for men’s singles is still based on the opponent’s mistakes (forced errors + non-forced errors + active errors). The percentage of mistakes leading to points in both formats exceeds 60%. However, from the specific breakdown of losing points, in singles matches, non-forced errors are more. This is consistent with the speculation mentioned earlier. In singles matches, there is a greater tendency toward risk gaming and more active offensive behavior, which can lead to more non-forced errors while obtaining more offensive opportunities. In both formats, active scoring is achieved through direct scoring and causing the opponent’s forced errors. The direct scoring rate in singles matches is slightly higher than that in team matches but does not have a significant difference. The forced errors in team matches are more and have a significant difference compared to singles matches. This can indicate that team matches are more inclined to control the game and seek opportunities to cause the opponent’s mistakes through pressure.

By analysing the scoring rates of various techniques in both formats, it can be seen that the kill and brush technique in individual matches has a much higher scoring rate than that in team matches. This shows that in singles matches, the offensive choices are more focused on maintaining a continuous downward pressure without making mistakes rather than winning with one shot. The pursuit of more stable scoring methods is more prominent. Other net-based techniques do not have significant differences in scoring rates. It can be seen that in men’s singles today, net-based techniques are more used as means to create offensive opportunities rather than as absolute means for direct scoring. Backcourt techniques are the main means for direct scoring. In men’s singles backcourt techniques, the most threatening techniques are the smash and the drop. There is no significant difference in the scoring rates of the smash in both formats. The scoring rate of the drop in individual event is higher than that in team event. As mentioned earlier, in the team event, the usage rate of drop is higher than in the individual event. More emphasis is placed on using drop to unsettle the opponent and avoid giving the opponent the opportunity to use clears. However, in the individual event, the scoring rate of drop is higher. It may choose to enhance the quality and speed to achieve the goal of scoring through drop.

The rate of losing points is similar in the two formats. Except for interception, other techniques do not show significant differences. Although the non-forced error rate of the individual match format is higher than that of the team match format in this indicator, the majority of the errors do not occur in the active offensive opportunities in the backcourt. This indicates that in the individual match format, athletes have a strong ability to create offensive opportunities and are very good at seizing the offensive opportunities, without the situation of high scoring rate and high losing rate resulting in low efficiency. The interception technique in the team event has a significantly lower rate of technical errors than that in the individual event. As one of the main means of passive overcorrection, interception has the characteristic of maintaining downward pressure. However, it has a high technical requirement. The interception technique is used more in the individual match format and has a higher rate of losing points. This can be explained by the fact that the scoring system of the team event has fewer points, and athletes use this technique better in the passive overcorrection type of movements.

### 4.5 Analysis of hitting routes and landing points

The research found that there are differences in the hitting routes and hitting line between the individual event and the team event. The hitting routes and hitting line to some extent reflect the application of athletes’ techniques and tactics and strategic choices. Some scholars have proposed that the application of techniques is closely related to the arc, line and hitting point of the ball, which is a technical characteristic of badminton (Zhang et al., 2007). There must be differences in the lines in the two formats. The connection between line selection and tactics and techniques is also very close. In both team and individual match formats, the most choices are in the backhand/top area, middle area, and forehand straight line. The hitting line of the straight line is the fastest and has the highest stability.

In the two formats, the landing points in the front court accounts for more than 50%. It can be seen that the control of the front court in men’s singles badminton matches is becoming increasingly important. Controlling the front court is the main means of initiating attacks. The differences in landing points between the individual event and the team event mainly lie in the left and right areas of the front court. The individual event has a higher landing point than the team event. This is because the team event uses less net-based techniques than the individual event. Although the number of drop shots used in the team event is higher than that in the individual event, it has little impact on the distribution of landing points in the front court. It can be seen that the individual event relies more on front court techniques, which is consistent with the previous statement that the individual match format uses more Spinning shuttle and net drop techniques. Other mid-court and back-court landing points have little difference. The only difference is the mid-court area in the backcourt, which is used more in the team event. In the sport of net-side holding, techniques can be divided into restrictive techniques and non-restrictive techniques. In the team event, when the ball is hit from the net-side area and the opponent is under pressure and starts to serve, more choices are made in the mid-court area. When lifting, choosing the left or right area can increase the distance that the opponent needs to move when attacking straight, but it will also cause the opponent to have a faster ball speed when attacking straight, bringing greater defensive pressure to the defenders. Choosing the mid-court area can make the opponent attack the area closer to the defenders when attacking straight, while maintaining the same distance when attacking straight. Therefore, in the team event, although choosing the mid-court area in the high ball serves does not bring the opponent’s relative pressure, it can improve the defensive efficiency and is in line with the tactical strategy proposed in the previous paragraph, which prioritizes “stability first.”

### 4.6 Characteristics of the 11-point scoring system in team events

The 11-point relay format is different from the previous team competition format. In addition to the first set where the players on the court can start the game regardless of the score, in the other sets, the players cannot start the game when the score is tied. They always face the situation of leading or lagging and start the game. Some studies have shown that in the game, due to the large disparity in strength between the two sides, both sides tend to play passively. For example, when one side has a large lead or a significant distance over the other side, the lagging side will not play with full effort because there is no possibility to catch up and overtake (Wang et al., 2013). The leading side will not play seriously because winning the game is already certain. In the 11-point relay format, the leading side will try to expand the lead advantage, while the lagging side, even when the score difference is too large, will try to catch up with the score. In conclusion, every point counts in the final outcome of the competition. Unlike in ordinary team events where the number of lost games does not affect the final result, every point does make a difference in this new competition format. Therefore, this new format has greatly reduced the occurrence of athletes playing negatively or not seriously in the competition. At the same time, it has narrowed the absolute difference in level between high-level and low-level athletes, and every point is crucial.

## 5 Conclusion

Following the adoption of the shortened format, the match pace shows a slight decline; however, the “every point matter” mechanism enhances competitive intensity, indicating that athletes must achieve rapid mental and physical readiness within limited time frames. The coexistence of 11-point and 21-point scoring systems offers event organizers two distinct formats: one supporting extended, strategic play and the other encouraging brief, high-intensity exchanges. This dual-format system improves the flexibility of tournament design, enabling organizers to meet diverse audience preferences. Furthermore, the reduced match duration may allow for more adaptable training cycle planning.

For individual events, training programs should emphasize net-control techniques, such as spinning net shots and lifts, to optimize high-risk, high-reward opportunities. In contrast, team event training should focus on minimizing unforced errors through tactics like drop shots and interceptions, along with exercises to build mental resilience. Given the condensed nature of the 11-point format, simulating high-pressure scenarios through short practice matches (≤15 min) can help improve athletes’ decision-making under fatigue.

## Data Availability

The original contributions presented in the study are included in the article/supplementary material, further inquiries can be directed to the corresponding author.
